# Post-traumatic right carotid-cavernous fistula resulting in symptoms in the contralateral eye: a case report and literature review

**DOI:** 10.1186/s12886-018-0863-6

**Published:** 2018-07-25

**Authors:** Linxin Zhu, Bing Liu, Jingxiang Zhong

**Affiliations:** 1 Department of Ophthalmology, The First Affiliated Hospital, Jinan University, 613 West Huangpu Ave, Guangzhou, 510632 China; 2 Ophthalmology Institute of Jinan University, Guangzhou, China

**Keywords:** CCF, Contralateral, Trauma, DSA

## Abstract

**Background:**

To report a case of a carotid-cavernous fistula (CCF) that occurred after a motor vehicle accident and review the uniqueness of this case and the main confusing points for the diagnosis of such cases.

**Case presentation:**

A 22-year-old man complained of left eyelid swelling, eye redness, visual decrease and occasional headache after motor vehicle accident 4 months prior during which he experienced a head injury. He was initially thought to have glaucoma, but he was finally diagnosed with a right CCF based on magnetic resonance imaging (MRI) and digital subtraction angiography (DSA). Timely embolization surgery resulted in obvious relief of the ocular symptoms and an improved prognosis.

**Conclusion:**

This is the first reported case of a post-traumatic unilateral CCF with contralateral symptoms in direct CCF, it is very infrequent and deserves our attention. We should maintain high suspicion of CCF and confirm the diagnosis by DSA when managing such patients to prevent serious consequences. Early diagnosis and treatment can improve the prognosis of patients.

## Background

Carotid-cavernous fistula (CCF) refers to an aberrant connection between the internal carotid artery (ICA), the external carotid artery (ECA) or any of their branches and the cavernous sinus (CS); it is rare but can cause serious consequences. The most common (70–90%) aetiology of CCF is trauma from a basal skull fracture. Uncommon causes include the spontaneous rupture of a cavernous carotid aneurysm or a pre-existing weakness of the arterial wall [1]. The CCF is typically divided into direct and indirect types according to the different arteriovenous shunts [2].Shunting of the blood from the carotid artery systems to the CS increases the pressure inside the CS, causes engorgement of the draining vessels, may cause the flow to reverse, and leads to myriad clinical manifestations that mimic many eye and neck diseases. Therefore, it is very important for neurosurgeons and ophthalmologists to focus greater attention on CCF. In our case report, this direct unilateral CCF with symptoms in the contralateral eye is very infrequent and easily misdiagnosed. To the best of our knowledge, this is the first reported case of a post-traumatic unilateral CCF with contralateral symptoms in direct CCF. Consequently, we think it is very meaningful to report this case and review more details about this disease.

## Case presentation

A 22-year-old man was referred to our hospital with complaints of left eye redness and swelling for more than a month. He had no history of nausea or vomiting, but he also complained of mild blurred vision, double vision and occasional headache for more than a week.

On further questioning, the patient revealed a history of a trauma. He was involved in a motor vehicle accident and received a head injury that involved basilar skull fractures and resulted in a subarachnoid haemorrhage and epidural haematoma. The patient received conservative treatment and was discharged from a local hospital following the alleviation of symptoms. However, the patient developed symptoms in the left eye 4 months after the injury. These symptoms included blurred vision, swelling, and hyperaemia of the left eye.

He denied a history of diabetes and hypertension. There was no history of pneumonia, tuberculosis, or any other infectious diseases. There was also no history of fever, sickness or any surgery. There was no loss of appetite or loss of weight. He was a non-smoker with no allergies to any medications.

**Fig. 1:**
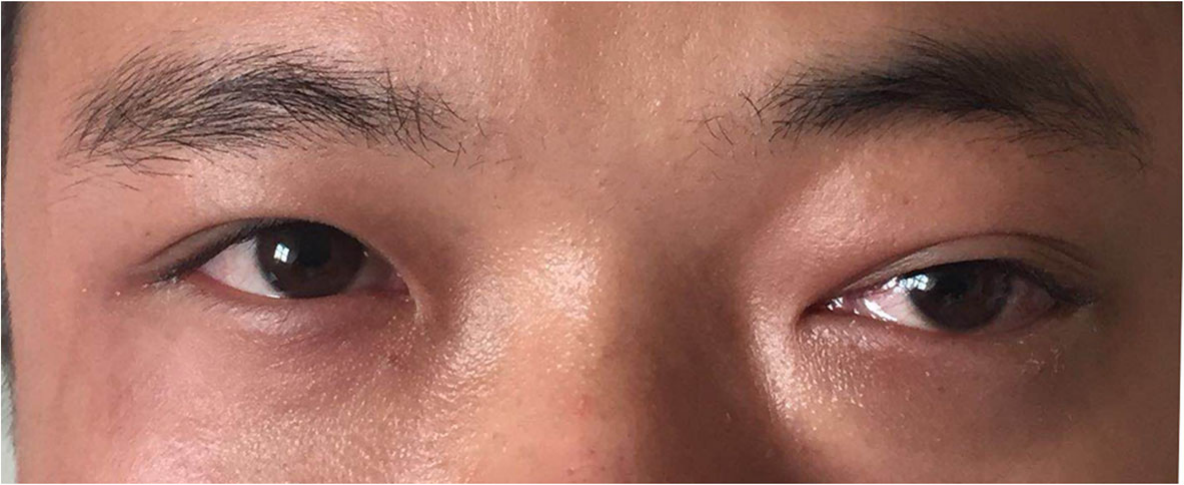
The appearances of both eyes. The right eye is almost normal, but the left eye exhibits slight eyelid swelling, chemosis, and hyperaemia

**Fig. 2:**
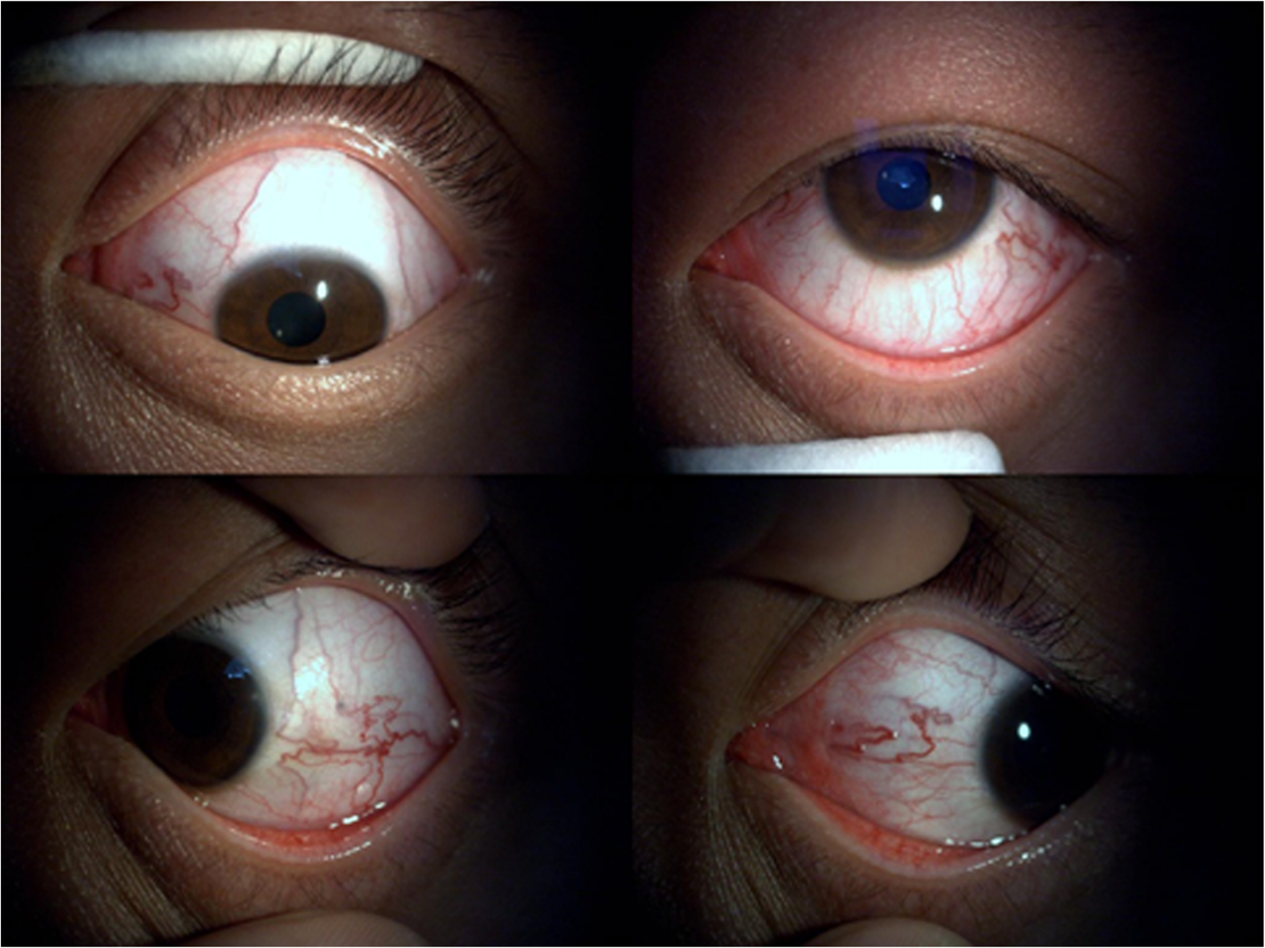
Slit lamp examination images of the four directions in the left eye revealed corkscrew hyperaemia centred on the cornea

**Fig. 3:**
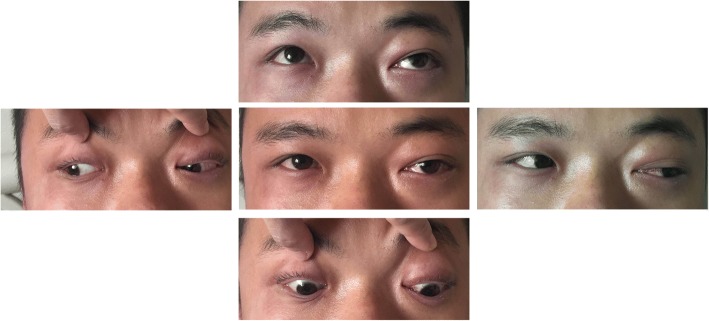
Extraocular muscle movement showed limitation on abduction and elevation of the left eye

**Fig. 4:**
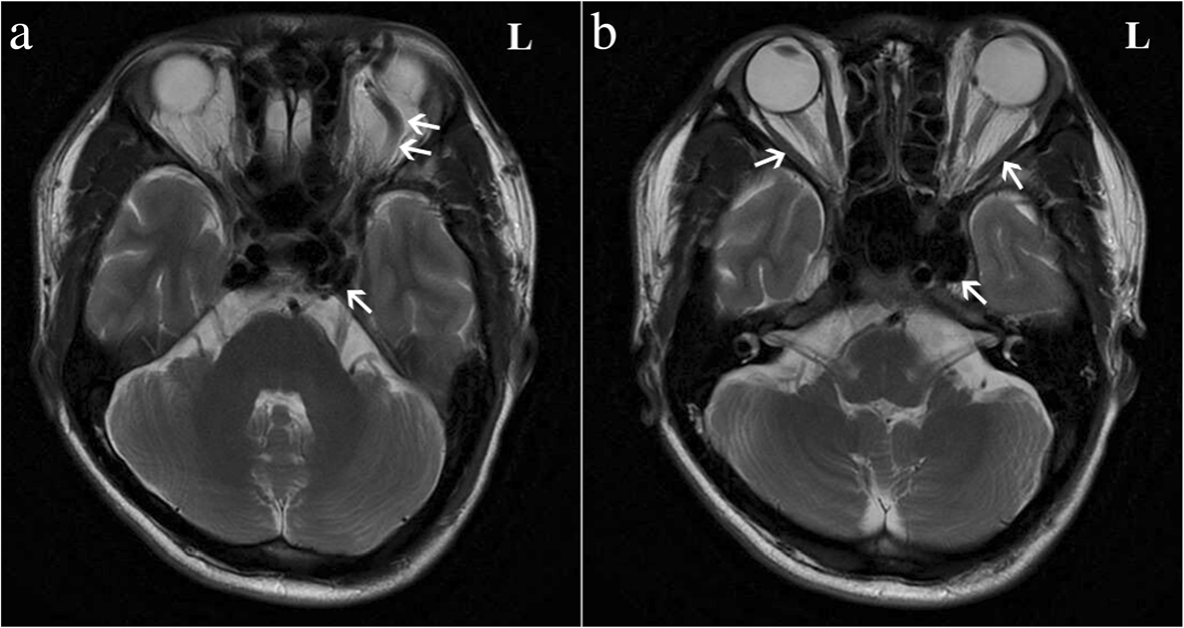
Magnetic resonance imaging (MRI) of the periorbital region. **a**. It revealed expansion of the left cavernous sinus and dilation of the left superior ophthalmic vein (white arrow). **b**. It revealed slight thickening of the left lateral rectus muscle and enlarged left cavernous sinus, yet the right was almost normal (white arrow).

**Fig. 5:**
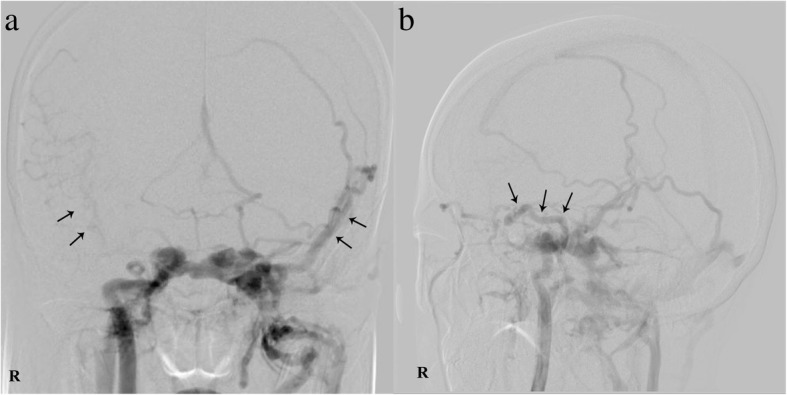
Digital subtraction angiography (DSA) images. **a**. Right cerebrovascular (CVA) filling delay (black arrow) and arterial blood traversing the intercavernous sinus resulting in expansion of the contralateral cavern. **b**. Broadening of the left superior ophthalmic vein (black arrow)

On examination, the visual acuity and intraocular pressure in the right eye of the patient were 6/5 and 17 mmHg, respectively, and the corresponding values for the left eye were 4/5 and 25 mmHg. On physical examination, there was no eyelid swelling, exophthalmos, ptosis or visual decrease of the right eye, and this eye was almost normal except for slight hyperaemia (Fig. 1). Extraocular muscle movement showed no limitation in the right eye (Fig. 3). However, the left eye exhibited eyelid swelling, mild ptosis, exophthalmos, chemosis, and corkscrew hyperaemia centred on the cornea (Figs. 1 and 2). Furthermore, there were some limitations of eye movement, and abduction and elevation of the left eye was − 1, yet movement on adduction and depression were normal (Fig. 3). The left anterior chamber was slightly shallow and quiet, but the right anterior chamber was normal. The cornea was clear with intact corneal sensation in both eyes, and there was no relative afferent pupillary defect and no anisocoria noted. The vitreous and lens were clear. Fundus examinations did not show disc swelling, obvious vascular dilatation or tortuosity, cotton wool spots or haemorrhages of either eye. The resident doctor previously doubted the presence of glaucoma, and he was admitted to our hospital. Optical coherence tomography (OCT) and field of vision tests did not reveal any abnormalities. By contrast, the MRI of the periorbital region revealed a broadening of the left superior ophthalmic vein, slight thickening of the left lateral rectus muscle, and an expansion of the left cavernous sinus, yet the right superior ophthalmic vein, extraocular muscles and cavernous sinus were almost normal (Fig. 4). These results aroused suspicion of the left CCF, so the patient was transferred to the neurosurgery department. The neurological examination was normal with the exception of a periorbital bruit on the left side. Thus, a tentative diagnosis of a left CCF was made. Surprisingly, cerebral angiography revealed a crevasse in the inner side of the intracavernous segment of the right internal carotid artery, the right cerebrovascular (CVA) filling delay, and arterial blood traversing the intercavernous sinus to reach the contralateral cavern, which resulted in the dilatation of the left ophthalmic vein (Fig. 5). Therefore, the patient was ultimately diagnosed with right CCF. Embolization surgery was suggested, but no additional treatment for the eyes was mentioned since most studies show that symptoms in the eyes could be completely relieved after aetiological treatment.

**Fig. 6:**
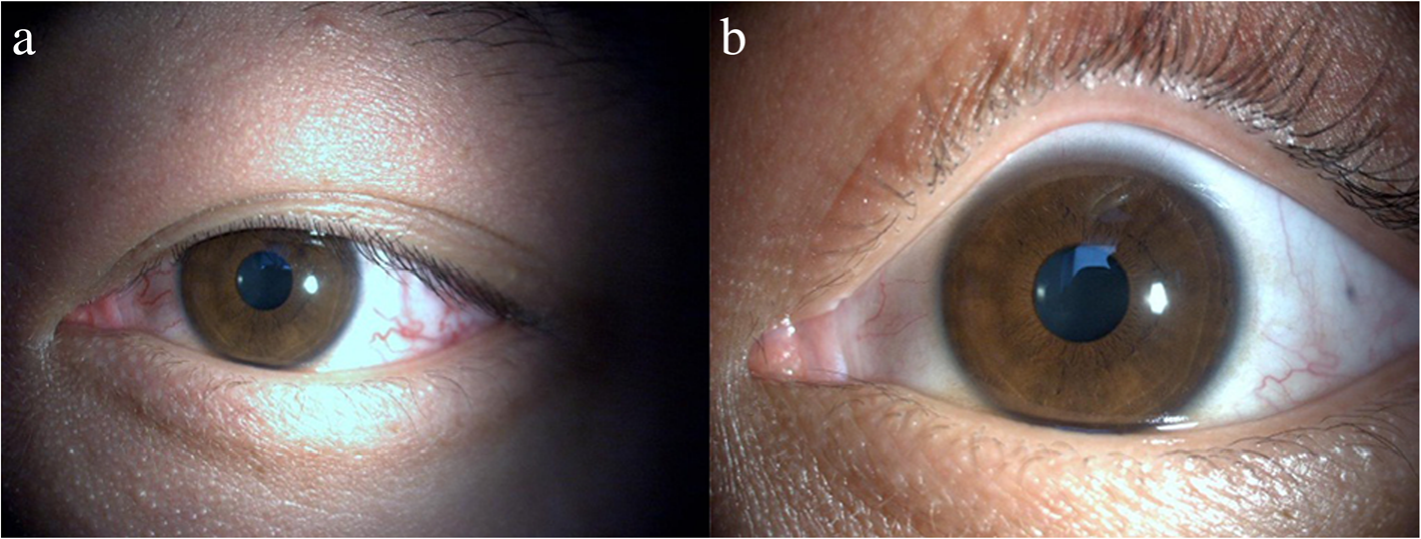
Following successful embolization surgery, the exophthalmos, chemosis and hyperaemia of the left eye were significantly relieved. **a**. The left eye before surgery. **b**. The left eye after surgery

Detachable balloon catheter embolization surgery was performed after several days. The surgery was successful, and the patient recovered well. All symptoms, including the redness and swelling of the left eye, the blurred vision, and the double vision, were resolved. On physical examination prior to the patient’s discharge, the visual acuity was improved to 5/5, and the intraocular pressure was 18 mmHg in the left eye, which was within the normal range. Additionally, the exophthalmos, chemosis and hyperaemia of the left eye were significantly relieved (Fig. 6).

## Discussion

### Aetiology and classification

CCF is a rare but lethal complication after trauma [3]. CCFs are reported to occur in 0.2% of patients with craniocerebral trauma and in up to 4% of patients with basilar skull fractures. Regarding aetiology, traumatic CCFs account for more than 70% of CCFs overall and are typically found in young male patients following closed head injuries [4, 5], as observed in the present case. The remaining 30% of CCFs occur spontaneously and are mostly observed in older female patients [6, 7]. Defects of the artery wall are thought to be responsible for the occurrence of CCFs following minor stress [4].

The CCF can be classified by their cause (spontaneous or traumatic), haemodynamic behaviours (high flow or low flow), and angioarchitecture (direct or indirect). Because the clinical manifestations of CCFs are closely related to the anatomical and haemodynamic characteristics of a fistula, Barrow and Peeters et al. [8] proposed a classification of CCFs into four types depending on the arterial supply in 1985, and this classification is preferred since it places more importance on the responsible blood vessels in the pathophysiology of CCF and has a therapeutic implication (as detailed in Table 1). Type A CCFs are the most common based on many studies; for example, Debrun GM et al. [7] classified 132 patients into the four types and found 75.8% type A, 3% type C, and 21.6% type D. In this report, the patient was a young male with an obvious trauma history (i.e., basilar skull fractures from a vehicular accident) prior to the presentation of the eye symptoms. In addition, DSA examination revealed a direct fistula between the right internal carotid artery and the cavernous sinus. Thus, according to Barrow’s classification, this direct unilateral CCF with symptoms in the contralateral eye belonged to type A. Nevertheless, Mario Zanaty et al. [9] concluded that in most circumstances, contralateral feeding vessels can exist only in indirect CCF (type B, C and D), so this case is infrequent. Additionally, only a few of the reported cases referred to contralateral flow into the carotid-cavernous fistula via the external carotid artery branches [10], but there was a scarcity of well-documented cases of exclusively contralateral direct carotid-cavernous fistulas with symptoms only present in the other eye. To our knowledge, this is the first reported case of a post-traumatic unilateral CCF with contralateral symptoms in direct CCF. Thus, this case report can considerably improve our experience in diagnosing such patients.

**Table 1:** The Barrow classification of CCFs

	Responsible blood vessel	Frequency	Etiology	Mainly Population	Haemodynamics	Progress
Type A	shunt between the ICA and cavernous sinus	More than 70%	associated with trauma	young male	high-flow CCFs	acute
Type B	shunt between the meningeal branches of the ICA and cavernous sinus	Less than 30%	Spontaneous	older female	low-flow CCFS	chronic or relapsing
Type C	shunt between the meningeal branches of the external carotid artery and cavernous sinus					
Type D	shunt between the meningeal branches of the ICA, external carotid artery, and cavernous sinus					

### Pathophysiology

The CS is located lateral to the sella turcica. The CS is a reticulated structure formed by an assembly of multiple thin-walled veins [11] and is the only structure in which the artery (i.e., the internal carotid) passes the vein network. When the intracavernous segment of internal carotid artery and the branches of the ICA or ECA are injured, the arterial blood flows directly into the CS through the fistula. The increased pressure in the CS results in a dilation in the ophthalmic vein (SOV and IOV). Subsequently, eye symptoms, including oedema, congestion, exophthalmos, increased intraocular pressure, among others, gradually emerge. By contrast, the relative perfusion pressure of the ophthalmic artery is simultaneously reduced, which causes retinal ischaemia and visual disturbances. Additionally, increased pressure within the CS can lead to the compression of its surrounding contents, which include cranial nerves (CN) III, IV, V and VI, and this compression can present as ophthalmoplegia [3]. In our case, the patient suffered from visual decrease and occasional headache, the left eye exhibited eyelid swelling, mild ptosis, exophthalmos, chemosis, corkscrew hyperaemia centred on the cornea and increased intraocular pressure (Figs. 1 and 2). Extraocular muscle movement showed slight limitation of the left eye (Fig. 3). In brief, all of the symptoms in the patient could be accounted for by the pathophysiology of CCF.

### Clinical presentation and differential diagnosis

The most frequent complaints involve the orbital region. The symptoms and signs of CCF always include eyelid swelling, proptosis, chemosis, and hyperaemia, and the condition is commonly misdiagnosed as Graves’ ophthalmopathy or inflammatory conjunctivitis. Ozlem Celik et al. [12] and Fausto Lore et al. [13] reported cases with both Graves’ disease and CCF in 2018 and 2003, respectively, and in both cases, the diagnosis of CCF was neglected. These authors concluded that if a patient presents with unilateral or asymmetrical eye symptoms or does not respond to the standard treatment, further examination is necessary. DSA can help to reach a final diagnosis. In the present case, the patient did not exhibit the symptoms of hypermetabolism or eyelid retraction and complained only about the left eye. Thus, we were able to exclude the possibility of Graves’ ophthalmopathy. Additionally, CCF and conjunctivitis can be differentiated by corkscrew hyperaemia centred on the cornea, and the patient had no infection history. Moreover, CCF can result in increased intraocular pressure, orbital pain, headache and blurred vision, which distinguish this condition from primary glaucoma. The characteristics of pulsating exophthalmos, orbital bruit and unilateral proptosis of more than 2 mm can aid the diagnosis to some extent, but imaging examination is necessary. Furthermore, the common treatment for glaucoma is not effective. In our case, the diagnosis of glaucoma was initially doubted; however, upon further questioning and examination, we quickly excluded this diagnosis. Moreover, if the cranial nerves are compressed by the engorged CS, ophthalmoplegia, diplopia and ptosis occur, and these symptoms can be easily confused with ocular myasthenia, especially in cases of indirect CCF with gradual progressive ocular symptoms. We can make this diagnosis via examinations of the acetylcholinesterase antibody, neostigmine experiments and thymus CT. In 2017, Lakshmi et al. [14] reported a case that was diagnosed as ocular myasthenia, underwent a thymectomy and was ultimately left with permanent hemiparesis. Thus, it is imperative to maintain a strong suspicion of CCF and to perform additional imaging examinations to avoid serious consequences. The patient in our case report was easily misdiagnosed because his symptoms were very slight and present in the contralateral eye. Therefore, to improve the prognosis of patients, the final diagnosis must be confirmed by DSA examination to ensure the correct and efficient treatment before the treatment or surgery is performed.

### Imaging examinations

When CCF is suspected for clinical reasons, the best initial tests are computed tomography (CT) or magnetic resonance imaging (MRI) since they are non-invasive, convenient and very quick. Many kinds of post-processing can visualize the proptosis, the cerebral oedema, and the cerebral haemorrhage. CT performs better for structures outside the blood vessels, especially bone fracture, yet MRI offers advantages of detecting flow voids as well as orbital oedema [3]. These methods can be helpful for primary judgement and provide more information for clinical diagnosis to distinguish other craniocerebral diseases. However, despite their ability to delineate certain draining veins, these methods rarely depict small feeding arteries in dural CCFs or the exact site of fistulous communication in direct CCFs. Therefore, these methods cannot replace conventional angiography, which is always needed for planning endovascular interventional treatment [4].

Recently, Alexander M et al. [5] and Hamm KD et al. [6] also concluded that CT and MR have a lower efficiency than DSA for the diagnosis of arteriovenous fistulas, which happen in cerebrovascular and microaneurysm in clinical research. DSA remains the gold standard for diagnosing CCF. It can not only help us diagnose CCF qualitatively but also detect the location and range of the nidus and provide detailed information on flow velocity, associated vascular injuries, and high-risk pathways since it can specially observe the blood vessels and detect haemodynamic processes. It can even reveal small dural feeding arteries that are missed on CT or MR. For example, Coskun et al. [4] reported a 69-year-old woman with a dural fistula that was missed on CT but finally diagnosed by the DSA result. In our case report, the direct fistula could not be detected on the MRI, which could only reveal some surface phenomena such as dilation of the left SOV and CS and slight thickening of the left lateral rectus muscle. However, the DSA identified the feeding vessel and the topography of the shunt, and the patient was diagnosed with right direct CCF. Thus, DSA is necessary for evaluating the angioarchitecture of the fistula, assessing the feeding arteries, and planning the intervention surgery [4]. Although DSA is an invasive test and has some complications such as thrombosis, cerebral vasospasm, nerve injury or haemorrhage, it is still the gold standard for diagnosing CCF.

### Treatment

The therapeutic methods for CCF treatment include conservative management, endovascular intervention, open surgery and radiosurgery. Higashida et al. [15] reported that conservative management can be effective in approximately 30% of indirect CCF and 17% of direct CCF cases. Interestingly, it has recently been reported that 20–60% of patients with indirect CCFs exhibit spontaneous fistula closure [16]. However, transarterial or transvenous embolization has remained the first-line treatment modality for most CCFs because over 90% of cases can be cured successfully in this manner [1, 17]. The patient in our case report was successfully cured with detachable balloon catheter embolization surgery; all of the symptoms were relieved without any sequelae or complications. Nevertheless, embolization of the CCF is associated with some risks that include thrombosis and reopening of the fistula. Thinda Sumeer et al. [18] reported a case of worsening angle closure glaucoma and choroidal detachments over an extended period of two months subsequent to the closure of a carotid cavernous fistula, and it has been reported that embolization through fragile and clotted veins can increase the risk of thrombosis [1]. Therefore, patients should be followed up, and their conditions should be monitored to avoid any subsequent risks after surgery.

## Conclusions

Most patients with CCFs initially present to ophthalmologists, and therefore, it is necessary for ophthalmologists to identify this condition by its characteristic clinical manifestations. Based on physical examination and medical history with special consideration of trauma, vessel and haemodynamic diseases should be considered so that a correct and timely presumptive diagnosis can be made. Additionally, the ophthalmologist should be able to order the appropriate tests to facilitate the diagnosis and monitor the course of the disease including extraocular changes, fundus changes and intraocular pressure (IOP) measurements [1].

Above all, the ophthalmologist should give special attention to CCF, ask detailed questions about the medical history, and conduct timely radiography examinations. In addition, to improve the prognoses of patients with CCF, the final diagnosis must be confirmed by DSA examination to ensure the correct and efficient treatment before the treatment or surgery is performed since a CCF on one side can result in symptoms in the contralateral eye. This situation is very unusual and cannot be detected by CT or MRI and merits our attention.

## Abbreviations

CCF: carotid-cavernous fistula
CN: cranial nerves
CS: cavernous sinus
CT: computed tomography
CVA: cerebrovascular
DSA: digital subtraction angiography
ECA: external carotid artery
ICA: internal carotid artery
IOP: intraocular pressure
IOV: inferior ophthalmic vein
MRI: magnetic resonance imaging
OCT: Optical coherence tomography
SOV: superior ophthalmic vein

## References

[1] Chaudhry IA, Elkhamry SM, Al-Rashed W, Bosley TM (2009). Carotid cavernous fistula: ophthalmological implications. Middle East African journal of ophthalmology.

[2] Ringer AJ, Salud L, Tomsick TA (2005). Carotid cavernous fistulas: anatomy, classification, and treatment. Neurosurg Clin N Am.

[3] Fattahi TT, Brandt MT, Jenkins WS, Steinberg B (2003). Traumatic carotid-cavernous fistula: pathophysiology and treatment. J craniofacial surgery.

[4] Ellis JA, Goldstein H, Connolly ES, Meyers PM (2012). Carotid-cavernous fistulas. Neurosurg Focus.

[5] Liang W, Xiaofeng Y, Weiguo L, Wusi Q, Gang S, Xuesheng Z (2007). Traumatic carotid cavernous fistula accompanying basilar skull fracture: a study on the incidence of traumatic carotid cavernous fistula in the patients with basilar skull fracture and the prognostic analysis about traumatic carotid cavernous fistula. J Trauma.

[6] Wang YW, Zhong Y, Ma J, Yang N, Wang KF, Jiang Y (2014). clinical features of carotid-cavernous sinus fistulas in 23 patients. Zhongguo yi xue ke xue yuan xue bao Acta Academiae Medicinae Sinicae.

[7] Debrun GM, Vinuela F, Fox AJ, Davis KR, Ahn HS (1988). Indications for treatment and classification of 132 carotid-cavernous fistulas. Neurosurgery.

[8] Barrow DL, Spector RH, Braun IF, Landman JA, Tindall SC, Tindall GT (1985). Classification and treatment of spontaneous carotid-cavernous sinus fistulas. J Neurosurg.

[9] Zanaty M, Chalouhi N, Tjoumakaris SI, Hasan D, Rosenwasser RH, Jabbour P (2014). Endovascular treatment of carotid-cavernous fistulas. Neurosurg Clin N Am.

[10] De Blasi R, D'Urso PI, Colamaria A, Occhiogrosso G, Ciappetta P (2010). Spontaneous carotid-cavernous fistula supplied by the contralateral meningohypophyseal trunk: case report and literature review. J Neurosurg Sci.

[11] Parkinson D (1995). Lateral sellar compartment: history and anatomy. J craniofacial surgery.

[12] Celik O, Buyuktas D, Islak C, Sarici AM, Gundogdu AS (2013). The association of carotid cavernous fistula with Graves' ophthalmopathy. Indian J Ophthalmol.

[13] Lore F, Polito E, Cerase A, Bracco S, Loffredo A, Pichierri P, Talidis F (2003). Carotid cavernous fistula in a patient with Graves' ophthalmopathy. J Clin Endocrinol Metab.

[14] Leishangthem L, Satti SR. Indirect carotid cavernous fistula mimicking ocular myasthenia. BMJ case reports. 2017;2017 10.1136/bcr-2017-222048 PMC5665180 29054956

[15] Higashida RT, Hieshima GB, Halbach VV, Bentson JR, Goto K (1986). Closure of carotid cavernous sinus fistulae by external compression of the carotid artery and jugular vein. Acta Radiol Suppl.

[16] Gemmete JJ, Chaudhary N, Pandey A, Ansari S (2010). Treatment of carotid cavernous fistulas. Curr Treat Options Neurol.

[17] Miller NR (2007). Diagnosis and management of dural carotid-cavernous sinus fistulas. Neurosurg Focus.

[18] Thinda S, Melson MR, Kuchtey RW (2012). Worsening angle closure glaucoma and choroidal detachments subsequent to closure of a carotid cavernous fistula. BMC Ophthalmol.

